# Neuropathic Pain and Related Depression in Mice: The Effect of a Terpene and a Minor Cannabinoid in Combination

**DOI:** 10.3390/biomedicines13123103

**Published:** 2025-12-16

**Authors:** Jose Rios, Mohammed Alnoud, Mohammad S. Hussain, Zayd Ayas, Emmanuel Franco, Justin Mills, Joshua Nwose, Maria Sophia Malbas, Hiram Garcia, Manish K. Tripathi, Khalid Benamar

**Affiliations:** 1 Institute of Neuroscience, Neuro & Behavioral Health ISU, School of Medicine, University of Texas Rio Grande Valley, Biomedical Building, McAllen, TX 78504, USA; jose.rios12@utrgv.edu (J.R.); mohammed.alnoud@utrgv.edu (M.A.); zayd.ayas01@utrgv.edu (Z.A.); emmanuel.franco01@utrgv.edu (E.F.); justin.mills01@utrgv.edu (J.M.); joshua.nwose01@utrgv.edu (J.N.); mariasophia.malbas01@utrgv.edu (M.S.M.); hiram.garcia03@utrgv.edu (H.G.); 2 Medicine and Oncology ISU, School of Medicine, University of Texas Rio Grande Valley, Biomedical Building, McAllen, TX 78504, USA; mohammad.hussain@utrgv.edu (M.S.H.); manish.tripathi@utrgv.edu (M.K.T.)

**Keywords:** beta-caryophyllene, cannabidiol, neuropathic pain, depression, drug combination

## Abstract

**Background/Objectives**: Neuropathic pain is one of the most severe types of chronic pain. Although it is difficult to manage, it often co-occurs with depression. Yet, no medication addresses the neuropathic pain and depression comorbidity. Therefore, developing integrated treatment strategies that address both pain and depression is a major public health priority and an unmet need affecting millions. **Methods**: In this study, we investigated the effect of combining a terpene, Beta-Caryophyllene (BCP), and cannabidiol (CBD) on neuropathic pain and associated depression. We employed a chronic constriction injury (CCI) neuropathic pain model and a series of behavioral tests to evaluate how oral administration of this combination influences neuropathic pain and depression-like behaviors in mice. We employed immunohistochemistry and proteomics approaches to explore the mechanism. **Results**: The analgesic effect of combining CBD and BCP is synergistic in neuropathic pain and also shows an antidepressant effect. Additionally, we found that this combination decreases neuroinflammation associated with CCI and affects specific genes involved in the inflammation. **Conclusions**: This work provides preclinical scientific evidence supporting the potential usefulness of this combination for neuropathic pain and associated depression.

## 1. Introduction

Chronic pain is a leading reason adults seek medical care [1], significantly affecting their quality of life [2,3,4,5]. Between 30% and 60% of individuals with chronic pain also experience depression [6,7,8,9], showing a strong connection between the two. Patients with both conditions face more severe and longer-lasting pain, and those with higher pain levels typically take longer to respond to treatment and recover from depression [10].

Neuropathic pain is now defined by the International Association for the Study of Pain as ‘pain caused by a lesion or disease of the somatosensory nervous system’ [11]. Neuropathic pain and depression are often treated with different medications, but the effectiveness of these treatments remains uncertain. The development of integrated therapeutic strategies for treating neuropathic pain while addressing depression is a significant public health priority and an unmet need.

Natural products have historically been sources of novel analgesic compounds developed into pharmaceuticals [12]. There is an increasing interest in exploring the medicinal potential of cannabis. Researchers have identified over 560 components within the cannabis plant [13]. Non-psychoactive cannabinoids like cannabidiol (CBD) are drawing significant attention for their therapeutic benefits. CBD, a minor cannabinoid found in cannabis, has emerged as a promising agent in recent years. Its appeal lies in its safety profile, as it does not produce the adverse central nervous system effects typically associated with cannabinoid receptor 1 (CB1), such as hypoactivity, hypothermia, and catalepsy [14].

The Food and Drug Administration (FDA) approved Epidiolex (CBD) oral solution for treating seizures associated with two rare and severe forms of epilepsy. CBD showed analgesic effects in several neuropathic pain rodent models with consistent results [15,16,17,18,19,20].

Despite the analgesic potential of CBD for pain, clinical trials to date have failed to demonstrate significant analgesic effects with pure CBD [21,22]. However, it has been suggested that cannabis compounds function more efficiently in concert with each other, rather than in isolation, a concept referred to as the “entourage effect” [23]. Preclinical data suggest that CBD may possess an antidepressant effect [24,25].

Cannabis plants contain various non-psychoactive compounds, including Caryophyllene (BCP) [26]. BCP is a natural bicyclic sesquiterpene that acts as a natural ligand for the cannabinoid type 2 receptor (CB2) [27] and is an FDA-approved food additive [28]. It has several benefits, such as pain relief, antidepressant effects, and anti-inflammatory properties [23,27,29,30,31,32,33,34,35,36].

Given this background, the main goal of this study is to test the hypothesis that combining CBD and BCP is effective for neuropathic pain while also demonstrating antidepressant effects.

Furthermore, we used immunohistochemistry (IHC) and proteomics approaches to explore the mechanism by which this combination produces analgesic and antidepressant effects in the CCI neuropathic pain model.

## 2. Materials and Methods

### 2.1. Animals

Mice (C57BL/6, male and female, 20–25 g, Jackson Laboratories, Bar Harbor, ME, USA) were housed in groups of five per cage, kept on a 12:12 h light-dark cycle, and had access to standard chow. All procedures received approval from the Institutional Animal Care and Use Committee at the University of Texas Rio Grande Valley.

### 2.2. Drugs

CBD and BCP were purchased from Cayman (Ann Arbor, MI, USA). Anti-Iba1 antibody and Anti-GFAP antibody were purchased from Abcam (Cambridge, UK).

### 2.3. Chronic Constriction Injury (CCI) Neuropathic Pain Model

The skin on the lateral surface of the right thigh was incised; a small incision was made through the biceps femoris to expose the sciatic nerve. Subsequently, four loose ligatures were tied proximally to the trifurcation of the sciatic nerve using silk sutures. The muscle and skin were closed, and the animals were monitored closely during surgical recovery. The surgical manipulations (CCI) were performed under deep general anesthesia (isoflurane, up to 4% for induction as needed; 1–2% for maintenance). The depth of anesthesia is assessed by regularly testing the corneal blink and hind paw withdrawal reflexes, as well as monitoring breathing patterns. Sterile procedures were used.

### 2.4. Behaviors

#### 2.4.1. Pain-like Behaviors

##### Mechanical Allodynia [37]

Mice were individually housed on an elevated wire mesh platform until exploratory behavior stopped. A semi-flexible filament (Digital von Frey method, IITC Life Sciences, Woodland Hills, CA, USA) was applied with increasing force (in grams) until a withdrawal response was observed. The force at which this response occurred was automatically recorded as the paw withdrawal threshold.

##### Thermal Hypersensitivity [38]

Animals were individually placed in Plexiglas boxes for acclimatization. A drop of acetone was applied to the plantar surface of each paw. The duration of paw elevation, licking, biting, and shaking in response to stimulation was recorded for 60 s.

##### Mouse Grimace Score (MGS) [39]

This assessment evaluates four components of rodent grimacing: orbital tightening or closure, ear position, nose bulging, and cheek bulging. Recorded over five minutes with two cameras, one at the front and one at the back, each component is rated on a scale from 0 to 2. A score of 0 indicates a normal, non-painful state, 1 reflects moderate perceived pain, and 2 signifies severe perceived pain. Mice are placed in plexiglass cages measuring 4.5 × 3.5 × 3.5 inches.

#### 2.4.2. Depression-like Behaviors

Tail suspension test. The mouse is gently suspended by its tail in a secure apparatus, allowing observation of its behavior without interference. During a five-minute recording session, the mouse’s movements and reactions are captured on video. The total time the mouse remains immobile is recorded.

Forced swim test (FST) [40,41]. A mouse was positioned in a clear, cylindrical container with a height of 55 cm and a diameter of 20 cm, which was filled with water. The mouse was permitted to remain in the water for a duration of 5 min. During this time, the amount of time the mouse spent immobile was recorded and subsequently analyzed using a video-tracking system developed by Noldus (Wageningen, The Netherlands).

### 2.5. Isobologram

An isobologram is used to evaluate the interaction between CBD and BCP [42]. In this graph, the effective dose at 50% (ED50) of CBD is plotted on the *Y*-axis, while the ED50 of BCP is on the *X*-axis. The points are connected by a diagonal line called the “line of additivity”. If the ED50 for the combination of CBD and BCP lies below this line, it indicates that the two compounds act synergistically, producing a greater effect than when used individually.

### 2.6. IHC

The paraffin-embedded brain tissues were sectioned at 10 μm. The primary antibodies used were anti-Iba1 at a dilution of 1:1000 and anti-GFAP at 1:250. The secondary antibody employed was MACH 4 (Polymer) Universal HRP-Polymer (M4U534H). Digital images were captured using the Panoramic Scanner version 2.2.0-MIDI, and the 3DHISTECH CaseViewer software v. 2.4.0.53492 was utilized for image analysis.

### 2.7. Proteomics

Frozen mouse brain tissues were ground into a fine powder using liquid nitrogen [43] and transferred to 1.5 mL tubes. Proteins were extracted with 400 µL lysis buffer supplemented with protease inhibitors, incubated on ice (30–60 min), sonicated, and centrifuged (14,000 rpm, 1 h, 4 °C). Supernatants were collected and stored at –20 °C. Protein concentrations were determined by the Bradford assay, and 100 µg from each sample was diluted to 100 µL in lysis buffer. Proteins were reduced, alkylated, and denatured (95 °C, 10 min), then digested overnight at 37 °C with Trypsin/Lys-C. Digestion was stopped with 50 µL stop solution, and peptides were labeled using TMT 10-plex reagents. Labeled samples were pooled, dried, desalted, and reconstituted in 0.1% formic acid. One microliter of each sample, including a HeLa quality control, was analyzed by the Delta-independent acquisition method on an Orbitrap Exploris 240 mass spectrometer (Thermo Scientific, Waltham, MA, USA) over a 120 min gradient. Raw data were processed in Proteome Discoverer (v2.5 and v3.1) using the TMT 10-plex workflow.

### 2.8. Statistics

One-factor analyses of variance (ANOVA) was employed. Significant differences were confirmed using appropriate post hoc pairwise comparisons. Furthermore, a *t*-test and a Mann–Whitney test were performed to compare the means of responses between two groups when a single variable was affected. Statistical analyses were conducted using GraphPad Prism 9 (GraphPad Software, San Diego, CA, USA). A *p*-value of 0.05 was designated as the threshold for significance.

## 3. Results

### 3.1. Dose–Response (D-R) of CBD and BCP

**Figure 1:**
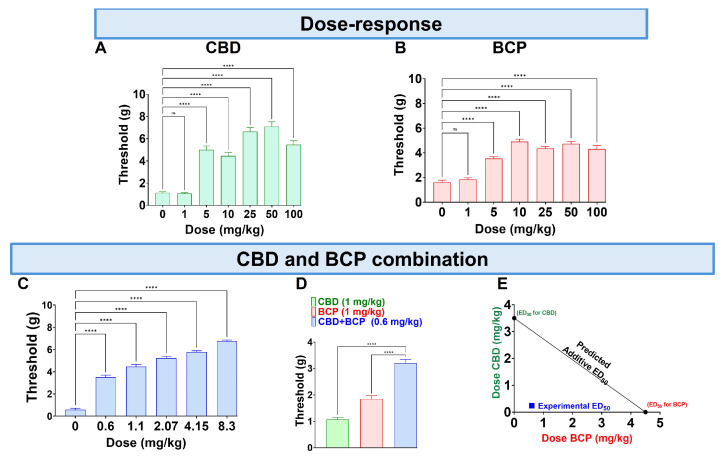
Administration of CBD (**A**), BCP (**B**), and their combination (**C**) on CCI-induced mechanical allodynia. The effects of CBD (1 mg/kg) and BCP (1 mg/kg) compared to the combination of CBD and BCP (0.6 mg/kg) on CCI-induced mechanical allodynia (**D**). CBD and BCP combined produce a synergistic effect (**E**). Data are shown as mean ± SEM. **** *p* < 0.0001. ns: not significant.

CBD administered orally (p.o.) at doses of 0.1–100 mg/kg produced a dose-dependent reduction in mechanical allodynia (Figure 1A, One-Way ANOVA with Dunnett’s Post Hoc Test, n = 10) with an ED50 of (3.5 mg/kg). The vehicle was olive oil.

The p.o. BCP administration at 0.1–100 mg/kg (p.o.) caused a dose-dependent decrease in mechanical allodynia (Figure 1B, One-Way ANOVA with Dunnett’s Post Hoc Test, n = 10) with an ED50 of 4.8 mg/kg.

Next, we tested the effect of the combination at a fixed-dose ratio of 1:1.4. The combination significantly reduced mechanical allodynia (Figure 1C, One-Way ANOVA with Dunnett’s Post Hoc Test, n = 5).

Interestingly, we found that CBD at 1 mg/kg and BCP at 1 mg/kg (Figure 1A,B) individually do not produce an analgesic effect. However, the combination of CBD and BCP at a lower dose of 0.6 mg/kg (0.2 mg/kg CBD + 0.4 mg/kg BCP) produces an analgesic effect (Figure 1D, One-Way ANOVA with Dunnett’s Post Hoc Test *p* < 0.0001, n = 5–10). These data indicate a synergistic effect. Additionally, the effect was confirmed to be synergistic through isobolographic analysis (Figure 1E). Finally, the ED50 of the combination is 0.84 mg/kg, which is lower than the ED50s for CBD (3.5 mg/kg) and BCP (4.8 mg/kg), showing that the combination is more potent than either CBD or BCP alone.

**Figure 2:**
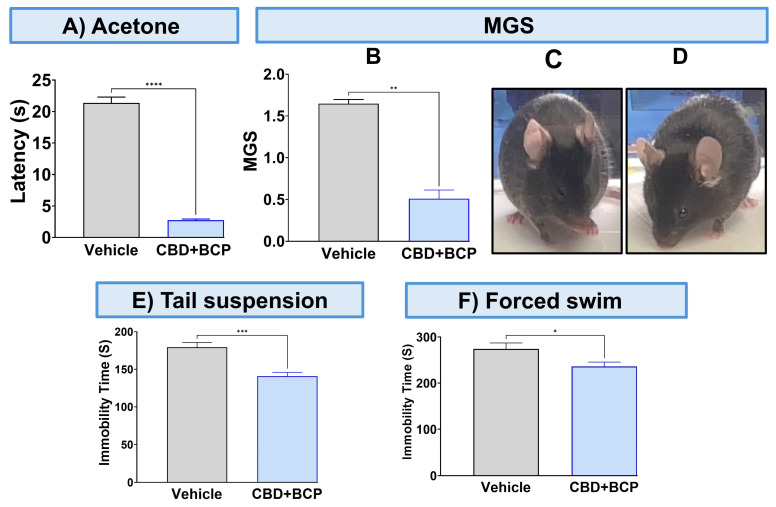
Effect of CBD and BCP in combination (8.3 mg/kg) in CCI-induced thermal hypersensitivity (**A**) and ongoing pain (**B**). Representative images of GMS from the vehicle (**C**) and CBD and BCP treatment (**D**). The effect of CBD and BCP in combination in the tail suspension (**E**) and the forced swim (**F**) tests. Data are presented as mean ± SEM. * *p* < 0.05, ** *p* < 0.01, *** *p* < 0.001, **** *p* < 0.0001. MGS, mouse grimace scale.

To validate the analgesic effect of this combination, we used other pain tests. The combination of CBD and BCP significantly attenuated thermal hypersensitivity (Figure 2A, *p* < 0.0001, *t*-test, n = 5) using the acetone test. The CBD and BCP treatment also significantly reduced MGS (Figure 2B, *t*-test, *p* < 0.01, n = 5). Representative images of the facial expression of CBD and BCP combination treatment (Figure 2D) show that this combination resulted in decreased nose and cheek bulges, eyes halfway closed, and ears positioned back, away from the face, compared to the vehicle (Figure 2C).

### 3.2. CBD and BCP Combination Produced an Antidepressant Effect in the CCI

The CBD and BCP combination was tested for depression-like behaviors using the tail suspension and FSTs. The administration of the CBD and BCP combination in mice with CCI significantly decreased immobility time related to chronic pain in the CCI in the tail suspension (Figure 2E, *t*-test, *p* < 0.001, n = 10) and FST (Figure 2F, *t*-test, *p* < 0.05, n = 5) compared to CCI mice treated with vehicle.

### 3.3. The Effect of CBD and BCP in Combination in Female Mice with CCI

**Figure 3:**
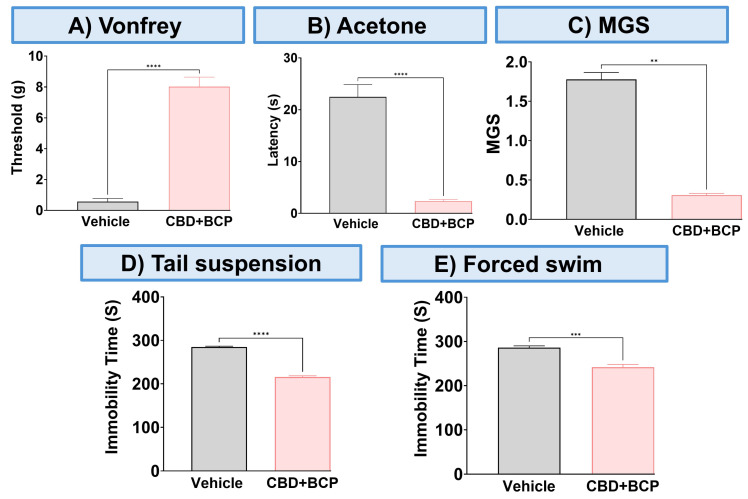
The effect of CBD and BCP in combination (8.3 mg/kg) on female mice with CCI. Effect of CBD and BCP in combination in CCI-induced mechanical allodynia (**A**) thermal hypersensitivity (**B**), ongoing pain (**C**), antidepressant using tail suspension (**D**), and FST (**E**). Data are presented as mean ± SEM. ** *p* < 0.01, *** *p* < 0.001, **** *p* < 0.0001.

**Figure 4:**
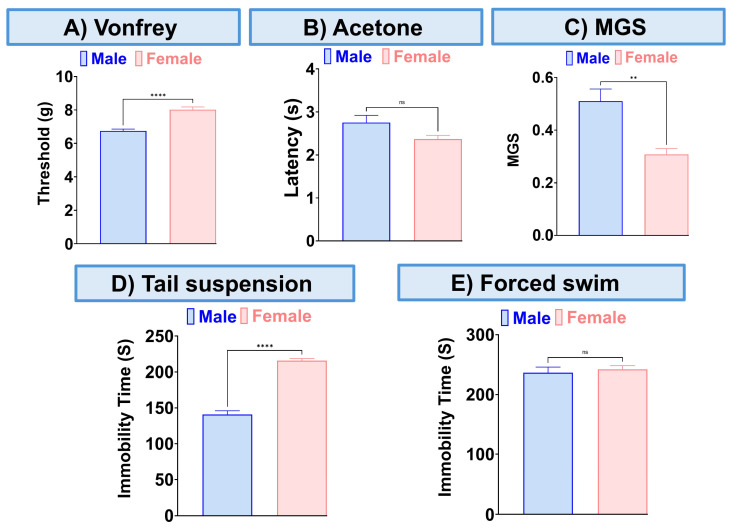
Sex differences in the effect of this combination (8.3 mg/kg) on mechanical allodynia (**A**), thermal hypersensitivity (**B**), MGS (**C**), and tail suspension (**D**), the forced swim test (**E**). Data are presented as mean ± SEM. ** *p* < 0.01, **** *p* < 0.0001.

The combination of CBD and BCP reduced mechanical allodynia (Figure 3A, *p* < 0.0001, *t*-test, n = *5*), thermal hypersensitivity (Figure 3B, *p* < 0.0001, *t*-test, n = 5), and ongoing pain (Figure 3C, *p* < 0.0001, *t*-test, n = 5). We also examined the effect of this combination on depression-like behaviors. The depression-like behaviors associated with neuropathic pain in the CCI model were significantly decreased by the CBD and BCP combination compared to the vehicle group in tail suspension (Figure 3D, *p* < 0.0001, *t*-test) and FST (Figure 3E, *p* < 0.001, *t*-test). Among the behavioral tests we performed (Figure 4) we found sex differences in the effect of this combination on mechanical allodynia (Figure 4A, *t*-test, *p* < 0.0001), MGS (Figure 4C, *t*-test, *p* < 0.01), and tail suspension (Figure 4D, *t*-test, *p* < 0.0001).

### 3.4. Effects of BCP and CBD Combination on Miscroglia and Astrocytes

For histological staining, we used two specific markers: ionized calcium-binding adapter molecule 1 (Iba1) for microglia and glial fibrillary acidic protein (GFAP) for astrocytes.

#### Effect of BCP and CBD Combination on Microglia

**Figure 5:**
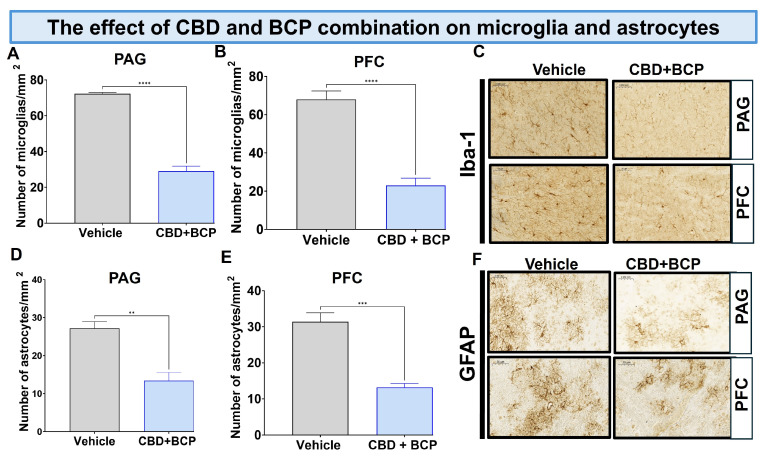
The CBD and BCP combination (8.3 mg/kg) reduced the expression of Iba1 in the PAG (**A**) and PFC (**B**) in the brain of CCI mice. Representative images of Iba1 in PAG and PFC (**C**) immunostaining in CCI treated with the combination of vehicle. The CBD and BCP combination (8.3 mg/kg) reduced the expression of GFAP in the PAG (**D**) and PFC (**E**) in the brain of CCI mice treated with the combination or vehicle. Representative images of Iba1 in PAG and PFC (**F**) immunostaining. These images are captured from the periaqueductal gray area. ** *p* < 0.01; *** *p* < 0.001, **** *p* < 0.0001; Data are presented as means ± SEM. N = 5 mice per group. Scale = 0.05 mm.

The CCI treated with vehicle resulted in a notable increase in Iba1 expression in the brain (Figure 5A,B, *t*-test, *p* < 0.0001, n = 5). The combination of CBD and BCP led to a significant decrease in Iba1 expression in CCI mice compared to the vehicle control in the periaqueductal gray (PAG) (Figure 5A; *t*-test, *p* < 0.0001, n = 5) and prefrontal cortex (PFC) (Figure 5B; *t*-test, *p* < 0.0001, n = 5). Representative sections of the PAG (key brain involved in descending pain modulatory system) and PFC (brain area involved in depression) (Figure 5C).

Effect of BCP and CBD combination on astrocytes. The CCI treated with vehicle resulted in a notable increase in GFAP expression in the brain (Figure 5D,E, *t*-test, 0.01, n = 5).

The CBD and BCP in combination produced a significant decrease in GFAP expression in mice with CCI in the PAG (Figure 5D, *t*-test, *p* < 0.01, n = 5) and PFC (Figure 5E, *t*-test, *p* < 0.001, n = 5). Representative sections of the PAG and PFC (Figure 5F).

### 3.5. Proteomics

#### 3.5.1. Heatmap of Differentially Expressed Proteins (Figure 6A)

Heatmap clustering analysis revealed distinct proteomic profiles between vehicle and the combination of CBD and BCP (n = 5). The rows represent individual proteins, and the columns correspond to the 10 samples analyzed. The expression patterns showed clear separation, with several protein clusters displaying either strong upregulation (red) or downregulation (green) in the combination of CBD and BCP group compared to vehicle control, indicating treatment-dependent proteomic remodeling.

**Figure 6:**
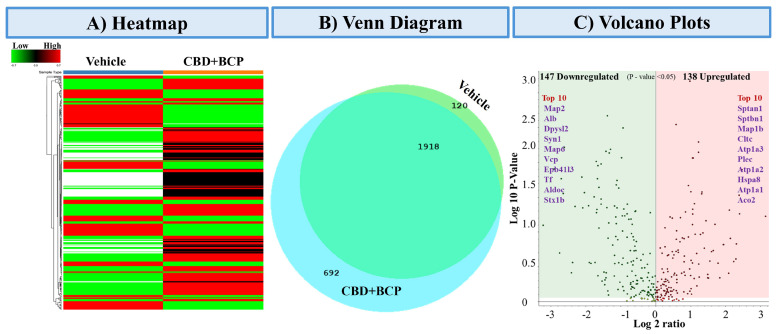
Proteomics analysis of the combination of CBD and BCP (8.3 mg/kg) effects. (**A**) Heatmap representing differentially expressed proteins comparing vehicle-treated and CBD + BCP-treated groups. Rows represent proteins, and columns represent experimental groups. Red indicates upregulated, green indicates downregulated, and black indicates unchanged expression. (**B**) Venn diagram showing the overlap and unique distribution of identified proteins between vehicle and CBD + BCP groups. A total of 1918 proteins were common to both treatments, while 692 were unique to CBD + BCP and 120 were unique to the vehicle. (**C**) Volcano plot summarizing significantly regulated proteins (*p* < 0.05). A total of 147 proteins were downregulated (green) and 138 proteins were upregulated (red) by CBD + BCP treatment. The top 10 upregulated and downregulated proteins are highlighted by name.

#### 3.5.2. Venn Diagram of Protein Overlap (Figure 6B)

Comparative proteomic profiling revealed both shared and unique proteins among the treatment groups. A total of 2730 proteins were commonly identified across conditions, while 120 proteins were unique to the vehicle group and 692 were unique to the combination of CBD and BCP. These results highlight both conserved and treatment-specific protein signatures.

#### 3.5.3. Differential Expression Volcano Plot (Figure 6C)

Volcano plot analysis identified 147 significantly downregulated and 138 upregulated proteins (*p* < 0.05) following the combination of CBD and BCP treatment. Among the most downregulated were Map2, Alb, Dpysl2, Syn1, and Map6, while the most upregulated included Sptan1, Sptbn1, Map1b, Cltc, and Atp1a3. These proteins represent potential molecular targets and pathways influenced by the combination of CBD and BCP.

#### 3.5.4. GeneMANIA Analysis of Inflammatory Genes (Figure 7)

To explore the molecular pathways affected by the combination of CBD and BCP treatment, we conducted GeneMANIA network analysis on inflammation-related genes identified in the brain. The combination of CBD and BCP exhibited denser, highly interconnected networks, suggesting improved coordination among inflammatory signaling genes. Key hub genes included Neuronal Pentraxin Receptor (NPTXR), Neuronal Pentraxin-2 (NPTX2), Serpin B6, Peroxiredoxi1 (PRDX1), IGFBP5, IGFBP6, Apolipoprotein E (APOE), platelet-type 6-phosphofructokinase (PFKP), and Oxysterol-Binding Protein 1 (OSBP).

**Figure 7:**
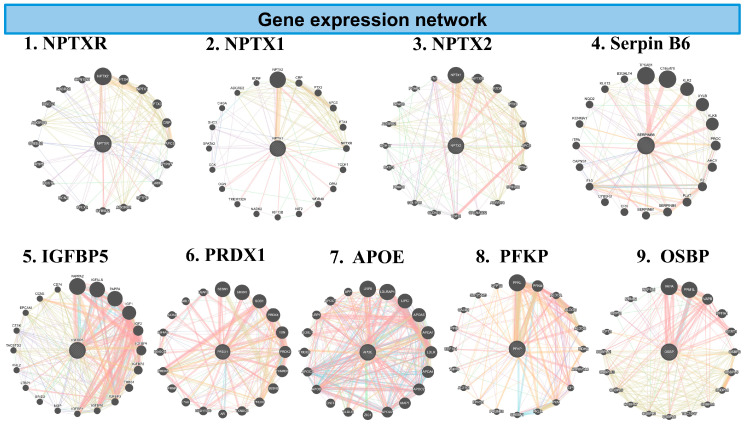
The network diagrams represent GeneMANIA analysis of inflammatory genes downregulated in the combination of CBD and BCP mouse brain tissue vs. control in proteomic analysis. Each circular plot shows gene–gene interaction networks of the inflammation-related gene. Nodes (circles): represent genes. Larger nodes indicate higher connectivity or importance within the network. Edges (colored lines): represent functional associations between genes. Different colors indicate the type of predicted interaction, including: co-expression, physical interaction, shared protein domains, genetic interaction, and predicted pathways. Central nodes: Highlight the query genes of interest (e.g., PRDX1, APOE) and their most functionally related partners. Peripheral nodes: represent genes indirectly associated with inflammation signaling.

## 4. Discussion

This study addresses two major and critical health conditions that affect millions: neuropathic pain and depression comorbidity. Addressing neuropathic pain alongside depression is a critical public health priority that remains unmet.

This study reveals two compelling findings: (1) the combination of CBD and BCP produced a synergistic analgesic effect in the CCI neuropathic pain model; and (2) this combination also exhibits antidepressant effects in the CCI neuropathic pain model, indicating that it can effectively relieve neuropathic pain while also addressing depression.

Neuropathic pain is one of the most severe forms of chronic pain. People with neuropathic pain often have hyperalgesia (decreased pain threshold), allodynia (extreme sensitivity to touch), and ongoing pain (sensations of pins and needles, shooting, burning, stabbing, and electric shocks). Current pharmacological treatments for neuropathic pain management, including gabapentin—the Gold standard—often demonstrate limited effectiveness and are associated with various side effects [44,45,46,47]. Furthermore, the frequent occurrence of comorbid conditions, such as depression alongside chronic pain [6,7,8,48], underscores the necessity for an integrative approach that addresses both issues. Better outcomes can be achieved by developing new and improved therapeutics or, more immediately, by identifying favorable compounds that are already available or emerging as potential new analgesics.

Our findings indicate that the combination of CBD and BCP attenuates CCI-induced neuropathic pain, and the effect was synergistic. Moreover, the ED50 of the combination is lower than the individual ED50 of CBD and BCP. This suggests that the combination of CBD and BCP is more potent than the individual effects of each.

Taken together, these data strongly support the concept of the entourage effect [23] and validate the potential of cannabis-based therapies in addressing pain.

Using other pain tests, this combination showed an analgesic effect in acetone (thermal hypersensitivity) and MGS (ongoing pain). This is important because one of the factors contributing to the high failure rate of analgesic drug candidates in clinical trials is the reliance only on mechanical or thermal stimulus-evoked behavioral outcomes, without addressing the ongoing pain aspect of chronic pain that is relevant to pharmacotherapy in humans. Addressing this issue is vital as it leads to a better and more accurate prediction of the efficacy of this combination in managing neuropathic pain resulting from sciatic nerve injury.

We also tested this combination for its potential beneficial effect on the depression associated with neuropathic pain induced by CCI. Our findings revealed that this combination exhibited a significant antidepressant effect, as demonstrated by improved depression-like behavior in two tests, tail suspension and FST. Therefore, we provide preclinical evidence in support of this combination as an integrated treatment strategy that addresses both neuropathic pain and depression.

We found that this combination effectively addresses sensory pain, ongoing pain, and depression in the CCI neuropathic pain model using female mice. When we compared the effects of this combination in male and female mice with CCI, we found sex differences in mechanical allodynia, MGS, and the tail suspension test. Specifically, this combination proved to be more effective in female mice for alleviating mechanical allodynia and ongoing pain. However, in terms of depression, it was more effective in male mice as demonstrated in the tail suspension test.

Given that inflammation is involved in pain and depression, we examined whether the beneficial effect of this combination in pain and depression involved an anti-inflammatory mechanism [49,50,51,52,53,54].

First, we found that the combined administration of CBD and BCP markedly attenuated Iba1 expression in the brain. Similarly, we evaluated the impact of this combination treatment on astrocytes, another critical cell type involved in neuroinflammatory processes. The combination treatment of CBD and BCP resulted in a notable reduction in GFAP density in the brain. Therefore, one possible mechanism by which this combination exerts analgesic effects is by attenuating the microglia and astrocyte expression induced by CCI.

Second, our proteomics data show that the combination of CBD and BCP alters the expression of inflammatory genes. The heatmap revealed a clear separation between the vehicle and the combination, highlighting the broad impact of the combination of CBD and BCP on inflammatory proteins. Similarly, the volcano plot identified a group of highly significant genes whose altered expression patterns may be associated with reduced inflammatory signaling.

GeneMANIA analysis shows the key inflammatory genes downregulated in the CBD and BCP combination, including NPTXR, NPTX2, Serpin B6, PRDX1, IGFBP5, IGFBP6, APOE, PFKP, and OSBP. These data suggest that this combination may reduce pain and depression by affecting these specific genes.

Although we performed proteomics analysis to identify key target genes, the functional significance of these genes in the effects of CBD and BPC combination on pain and depression requires further investigation in vitro and in vivo experiments.

In conclusion, the proposed project introduces the concept that the combination of CBD and BCP can effectively relieve neuropathic pain while also addressing depression. This knowledge will advance the field by providing preclinical scientific evidence supporting the potential usefulness of this combination for neuropathic pain and associated depression.

## Data Availability

The original contributions presented in this study are included in the article. Further inquiries can be directed to the corresponding author.
